# Supplementary material for: Hippocampal and medial prefrontal cortices encode structural task representations following progressive and interleaved training schedules
Source: PLoS Comput Biol. 2022 Oct 17;18(10):e1010566. doi: 10.1371/journal.pcbi.1010566 (PMC9612823; doi:10.1371/journal.pcbi.1010566)
Supplement: S1 Table — Rounded parentheses represent bootstrapped standard errors and square braces indicate bootstrapped 95% confidence intervals. (PDF) [file pcbi.1010566.s004.pdf]

S1 Table

	Interleaved training			Progressive training	
	Recent	*Remote*		*Recent*	*Remote*
AND model	-.004<br>(.104)<br>[-.191, .219]	.067<br>(.092)<br>[-.120, .239]		.096<br>(.105)<br>[-.138, .277]	-.230<br>(.074)<br>[-.364, -.070]
OR model	.462<br>(.107)<br>[.223, .647]	.545<br>(.127)<br>[.178, .731]		.543<br>(.074)<br>[.354, .658]	.317<br>(.117)<br>[.043, .514]
