# Supplementary figures and images for: Hippocampal and medial prefrontal cortices encode structural task representations following progressive and interleaved training schedules
Source: PLoS Comput Biol. 2022 Oct 17;18(10):e1010566. doi: 10.1371/journal.pcbi.1010566 (PMC9612823; doi:10.1371/journal.pcbi.1010566)

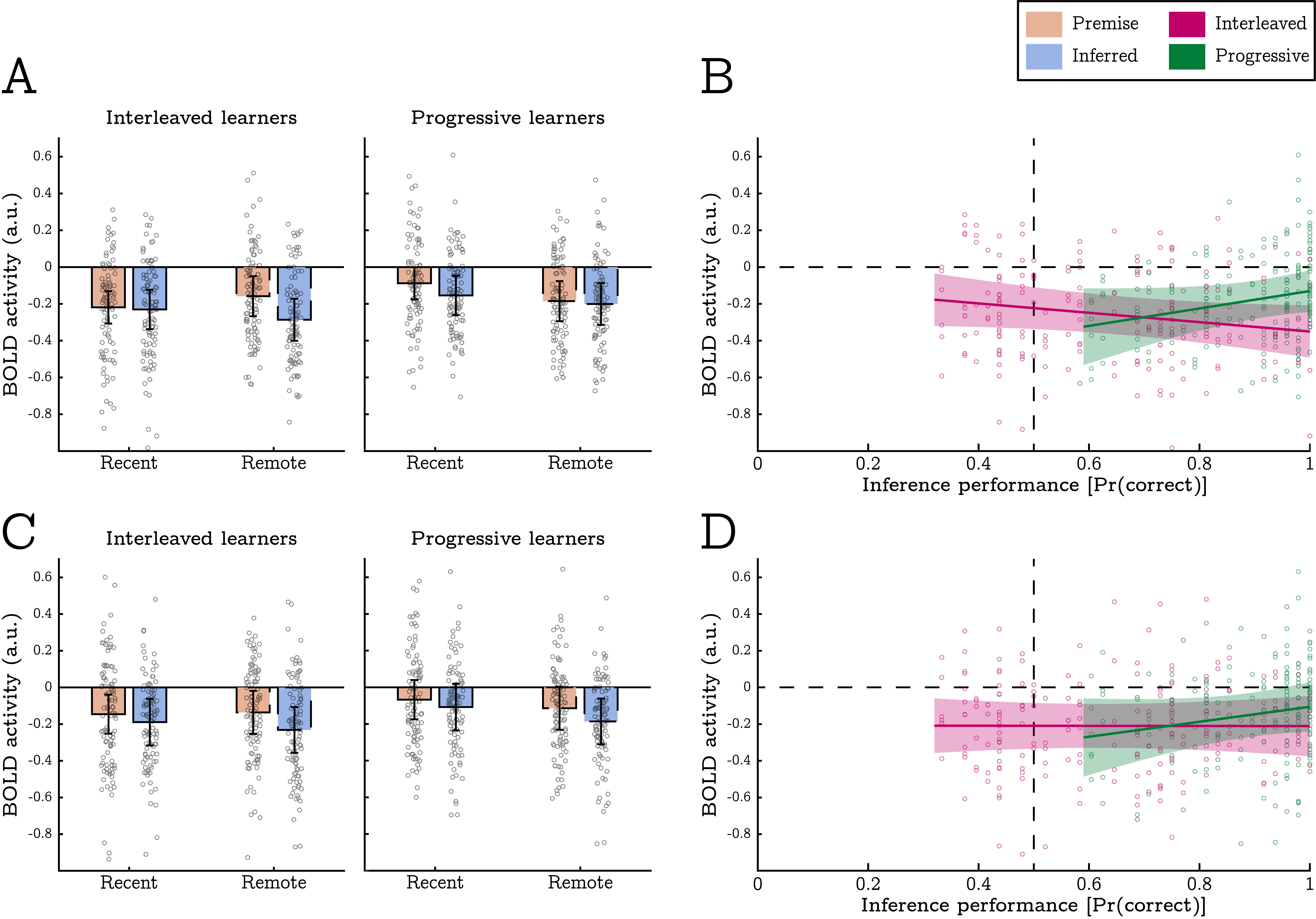

Supplement: S1 Fig — Panels A and B show activity in the left hippocampus. Panels C and D show activity in the right hippocampus. Bar charts display mean response amplitudes to all in-scanner discriminations split by trial type (premise vs inferred) and experimental condition (training method and session). Scatter plots display mean response amplitudes to all inference trials (both recent and remote) as a function of inference performance, split by training method (interleaved vs progressive). The only effect that reached statistical significance in these regions was detected in the right hippocampus. Here, a main effect of trial type indicated lower levels of BOLD activity on inference trails (panel C), yet this effect was not modulated by training method or inference performance. Individual data points indicate discrimination-specific BOLD estimates for each participant and error-bars indicate 95% confidence intervals. (TIF) [file pcbi.1010566.s005.tif]

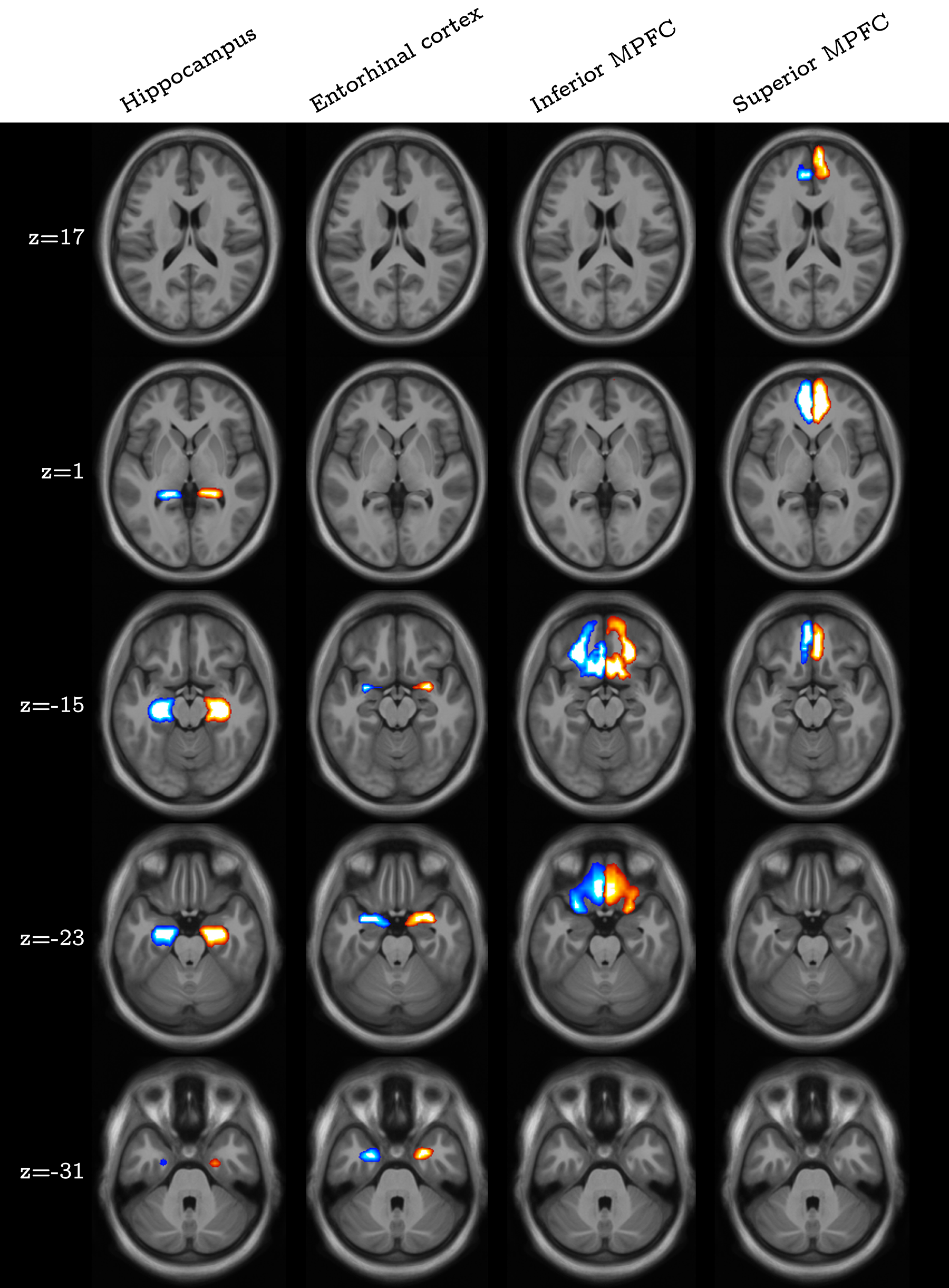

Supplement: S3 Fig — Each column relates to a different brain region and the blue-/orange- coloured overlays depict left-/right- hemisphere ROIs (respectively). Overlay lightness represents ROI coverage across participants. (TIF) [file pcbi.1010566.s007.tif]
