# Supplementary material for: Hippocampal and medial prefrontal cortices encode structural task representations following progressive and interleaved training schedules
Source: PLoS Comput Biol. 2022 Oct 17;18(10):e1010566. doi: 10.1371/journal.pcbi.1010566 (PMC9612823; doi:10.1371/journal.pcbi.1010566)
Supplement: Attachment — Submitted filename: Berens-Bird_2022_ResponseToReviewers_R3.pdf [file pcbi.1010566.s008.pdf]

We thank the reviewers who provided feedback on this manuscript, all of which were extremely constructive. We have endeavoured to address each point that was raised as fully as possible and feel that the amended manuscript is much improved in quality. Below we have copied each of the reviewer comments along with are our responses (in bold), and quoted sections of manuscript (indented).

Reviewer 1

This paper uses behavior, modeling, and fMRI to explore how transitive inference is impacted by order of presentation of information and by time. Premise pairs in a transitive hierarchy were either presented in progressive order or interleaved, and behavior and neural representations were investigated immediately after learning and after a 24 hour delay. Transitive inference performance was better after progressive relative to interleaved training and stronger for more distant pairs in the hierarchy, indicative of encoding- rather than retrieval-based mechanisms of inference. The medial temporal lobe and medial prefrontal cortex contained representations reflecting the hierarchical structure, including representations in the medial prefrontal cortex that appeared to generalize across distinct hierarchies. Results did not differ substantially as a function of time since learning.

The paper contributes an interesting investigation of the mechanisms of transitive inference from several methodological angles. The behavioral results are convergent with prior findings and the fMRI results suggest intriguing mechanisms of structure representation. However, as outlined below, I do not see the behavior and modeling as especially groundbreaking relative to prior work, and I have methodological concerns that dampen my enthusiasm about the imaging results.

We thank the reviewer for their extremely constructive input. We hope that our amendments and accompanying responses are sufficient to alleviate the reviewers concerns about our analysis methodology, and the theoretical implications of our findings.

1. The paper does not engage with a large prior behavioral literature on mechanisms of transitive inference, including an especially relevant sub-literature on order effects. For example, a recent study by Markant 2021 in Memory & Cognition assesses how the order of premise pair presentation affects explicitness of hierarchy knowledge. This paper contains citations to other papers on order manipulations. My sense is that there has been agreement that "chaining" the order of premise pair presentation, very similar to the progressive condition here, tends to result in better inference. The Markant study shows that chaining leads to more explicit awareness of the hierarchy. It seems likely that something similar could be going on in the progressive condition in this study: progression presentation leads to more explicit awareness of the hierarchy, and therefore to better inference performance, and performance that exhibits the distance effect. If the authors collected any post-study awareness questionnaires, that might be useful to analyze along these lines. Overall, this prior literature reduces the novelty of the main behavioral results (Figure 3) showing that progressive trial order results in better inference. There is also a smaller literature on the neural substates of transition inference that was not sufficiently discussed.

We thank the reviewer for highlighting these studies. We have now substantially revised large parts of the introduction and discussion sections to include the reports by Markant (2020/2021), along with several others that were either previously unknown to us or have been published very recently. Nonetheless, we disagree that the findings by Markant and others reduce the novelty and importance of our results for one key reason. Previously, it was unclear what led to the benefits of chaining. Even if such a training schedule increases explicit awareness of the hierarchy, the underlying reason for this could be attributable to a facilitation of either encoding- or retrieval-based mechanisms. We now make this point explicit in the introduction (page 3):

Despite this, it remains unclear whether and how interleaved vs blocked training influences the structure of memory representations that are learnt, or the generalisation mechanism that are preferentially employed. Behavioural evidence suggests that blocked training enables the learning of low-dimensional (compressed) stimulus representations that linearly encode task-relevant features [27]. Additionally, recent research shows that training overlapping discriminations in an ordered sequence (e.g., A>B followed by B>C and then C>D, so-called 'chaining') may improve transitive inference by allowing learners to integrate the discriminations into a unified mental model [29,30]. Nevertheless, when this integration takes place, and whether it depends on encoding- or retrieval-based mechanisms has yet to be tested.

2. I have mixed feelings about the modeling work in the paper. Given the one hidden unit, the only way the MLP model can solve the task is with a one dimensional value hierarchy. The model can only use an encoding strategy, and only this particular strategy. Still, it is a nice demonstration that progressive presentation can help establish the value hierarchy. I am not sure what is added by the AND and OR models. My understanding is that these are basically just evaluating whether there is a distance effect, and I am not sure if the Figure 4 results tell us anything new beyond Figure 3 B and D. Overall, the behavioral and modeling effects are nicely consistent with prior transitive inference behavioral work on distance effects and training order effects, but it is not clear that there is anything particularly new here.

We thank the reviewer for these comments as they have helped develop our thinking with regard to how we analyse, present, and discuss the results of our modelling work. As the reviewer mentions (and as echoed by Reviewer 3), the purpose of the MLP analyses is only to demonstrate that some neural networks do learn differently depending on whether they are trained via either progressive or interleaved training schedules. As a result of the reviews, we have now extended our analyses to include MLPs that have more than one hidden unit. Furthermore, we now discuss our results in the context of recent work examining how architectural differences affect generalisation (e.g., Flesch, Juechems, Dumbalska, Saxe & Summerfield, 2021).

In regard to the AND/OR models, the reviewer is correct that these analyses are principally designed to test for the presence of distance effects. However, rather than simply examining the strength of the correlation between transitive distance and performance, these models are explicitly formulated to account for difference in performance between premise pairs that could confound the results or reduce levels of statistical sensitivity. Indeed, on the basis of the correlations in Figure 3 alone, we are unable to adjudicate between encoding- and retrieval-based models of generalisation since all but one of the slopes are not significantly different from zero. Furthermore, we were motivated to use these models to test our strong a priori prediction: that different training schedules would bias the use of certain generalisation mechanisms – a prediction that we are able to strongly refute.

3. There is more potential for new mechanistic insight in the imaging results. The results seem promising and intriguing. But I was concerned that representations of individual items were inferred from trials where pairs of items were presented (as opposed to trials with individual items presented). My understanding is that in addition to the 6 premise pairs, the following inference trials were presented in the scanner: B>D, B>E, B>F, C>E, C>F, D>F. B and F are more distant in the hierarchy and presented more frequently, and I am worried that different frequencies of presentation could introduce potential confounds. For example, it could lead to different degrees of item-specific adaptation, which might affect the estimates of the individual item representations and the interpretation of both the within and across hierarchy similarities. Or the item-separation procedure could behave differently (exhibit different accuracy or different degrees

of differentiation from other items) for the B and F items, which are presented with three different other items, versus the C, D, and E items, each only presented with two other items.

Here, the reviewer raises some important questions regarding the validity of our pattern similarity analyses. We fully appreciate these points and so have substantially modified our analysis pipeline to ensure that all the reported effects cannot be attributed to the potential confounds highlighted by the reviewer.

First, given the above comments, we felt it was unsafe for the RSA to include any of stimuli at extreme ends of the transitive hierarchies (i.e., A and G in either the recent or the remote conditions). This is because, unlike all other stimuli, these 'terminal' stimuli were only presented in the premise discriminations. As such, their estimated BOLD representations may be distinct for reasons associated with general differences between premise and inferred trials. To address this, we simply removed all pattern similarity comparisons that involved the terminal stimuli.

Secondly, following a suggestion by Reviewer 2, we adapted the linear decomposition procedure to eliminate the assumption that BOLD patterns evoked on each trial are not influenced by overall differences in activity between premise and inferred trials. To do this, we included a term in the linear decomposition procedure that accounted for between trial-type differences. This effectively decreased the variance in the pattern similarity estimates by removing signals attributable to the different trial-types.

Finally, when controlling for nuisance effects in the linear mixed-effect models, we included an extra regressor that accounted for the temporal proximity of trials during the in-scanner task (i.e., adaptation effects), and correlations introduced by the pattern decomposition procedure itself. This regressor was added alongside another that controlled for shared sources of noise across co-presented wall textures. Full details are now included in the methods section on page 30:

> *Critically, the temporal structure of the in-scanner task and least-squared decomposition procedure introduced nuisance correlations between wall texture representations. To account for these, we derived two predictor variables of no-interest and used them to model nuisance effects in each LMM described below. The first predictor accounted for trivial correlations resulting from shared sources of noise across co-presented wall textures. This was taken as the Fisher-transformed correlation between binary vectors encoding whether each pair of wall textures were presented in same the in-scanner trails. Across analyses, this predictor invariably accounted for a significant amount of variance in the similarity scores,* $r \in [0.105, 0.277]$.
>
> *The second predictor of no-interest modelled nuisance correlations attributable to the temporal proximity of trials during the in-scanner task and the pattern decomposition procedure itself. To estimate these correlations, we simulated independent, normally distributed voxel patterns for all wall textures across a large number of iterations, mixed them together in accordance with the trial timings for each subject, and re-estimated the voxel patterns using the least-squares-separate decomposition procedure outlined above. The predictor of no-interest was then taken as the mean Fisher-transformed correlation between simulated wall-textures across iterations. Using this procedure, we aimed to model the effect of fMRI repetition suppression by parametrically modulating the simulated BOLD responses such that repeated presentations evoked an attenuated response. This adjustment was based on Fritsche*

et al. (2020) who report that repetition suppression effects in the parahippocampal cortex yield a BOLD attenuation of approximately 23% following a 100ms delay, and 10% following a 1 second delay. Given this, we applied repetition suppression effects assuming an exponential recovery from adaptation over time. Our simulations showed that the presence of such effects did not notably bias BOLD pattern recovery. Furthermore, the temporal signal-to-noise ratio of the fMRI signal had little effect on the correlational structure of the recovered BOLD patterns. Nonetheless, the simulations did reveal some minor nuisance correlations that tended to account for a significant amount of variance in the pattern similarity scores, $r \in [0.001,\ 0.183]$.

With regards to the reviewer's specific concern that the linear decomposition procedure could have been less accurate for certain wall-texture stimuli, we explicitly tested this using the stimulation referenced above. Here, we measured decoding accuracy by comparing corresponding and non-corresponding correlations between ground-truth BOLD representations and those estimated from the decomposition procedure. This highlighted negligible differences in the decoding accuracy of stimuli across a range of temporal signal to noise ratios as illustrated below (y-axis values depict the difference between correlations and error bars represent +/- 1 standard deviation across participant design matrices):

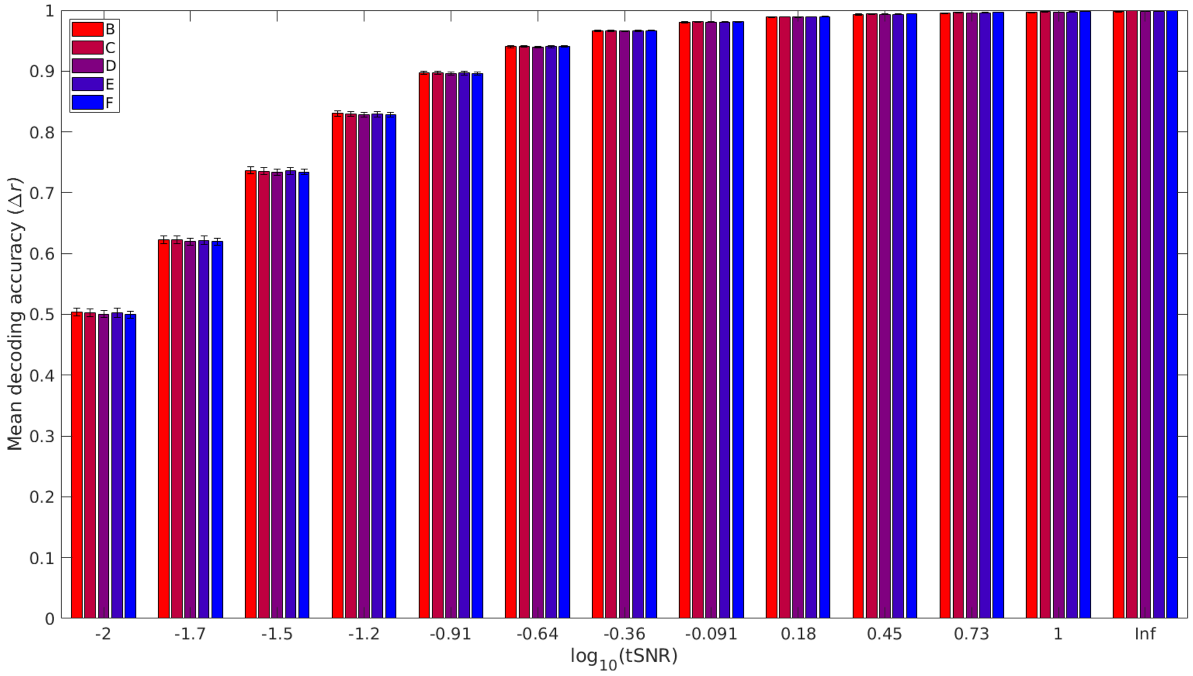

Reviewer 2

This study examines how knowledge of general structure (here, a hierarchy of the value of different items) is formed based on individual learning experiences. The study attempts to clarify the mechanisms involved using models of both learning and inference. Modeling of the learning phase suggests that progressive learning, compared to interleaved learning, may substantially improve learning of the underlying hierarchical structure of the task. Modeling of the retrieval phase suggests that participants can infer the relative ranking of different items in the hierarchy without having to remember the intervening items and their rank. The study also finds that hippocampus, entorhinal cortex, and medial prefrontal cortex represent the item hierarchy, given that the similarity of different items decreases as the distance between them in the hierarchy increases. Medial prefrontal cortex representations have increased similarity after progressive learning, suggesting that progressive learning may lead to each item being associated with a common representation of the hierarchy rather than separated item representations.

The study examines a number of factors that have been proposed to shape learning of general structure, including learning schedule, delay, and pattern similarity within hippocampus, entorhinal cortex, and medial prefrontal cortex. Examining these factors together helps clarify the contribution of each factor and their interactions to inference performance. It also uses modeling to help clarify some of the learning and retrieval mechanisms that may be involved in successful inference.

The perceptron model of the training phase helps to illuminate important effects that the learning schedule may have on formation of knowledge of item hierarchies. This extends prior work on effects of learning schedules on other cognitive tasks such as category learning, suggesting that the learning schedule should be matched to the structure being learned.

The statistical analysis is rigorous, taking many important factors into account and providing a strict control for multiple comparisons across brain regions.

A comprehensive code archive has been made publicly available and is well-integrated into the paper, with links to relevant sections. This makes the methods used substantially more replicable.

We thank the reviewer for their highly constructive comments a hope that our amendments and accompanying responses address all their concerns.

Major recommendations

1. The general linear model used to estimate the pattern of neural activation associated with each item relies on an implicit assumption, namely that activation during each item pair is determined based on the linear combination of activations associated with the items within that pair. However, analyses of univariate activation suggest that this assumption is violated; there are sometimes differences in activation between the premise and inferred pairs. This concern about the accuracy of the modeling of individual items also applies to the pattern similarity analyses, which rely on accurate estimation of the individual item pairs. Run a follow-up pattern analysis to address the concern that the general linear model of the individual scanned pairs does not take item interactions into account. If you just estimated the activation of each of the premise pairs, then you could examine their similarity as a function of distance without having to assume that they are formed through linear combinations of item patterns. Alternatively, you could attempt to control for differences between premise and inference pairs in the model used to estimate individual pair patterns; then you could estimate individual items from there. In this case, controlling for other factors such as response time and transitive distance might also help address other potential sources of variance.

We thank the reviewer for highlighting these important points. As noted in our response to Reviewer 1, we have substantially modified our analysis pipeline to ensure that all the reported effects are not attributable to potential confounds. Our principal hypotheses explicitly concerned neural representations of specific stimuli and so we chose to eliminate the assumption that BOLD patterns are not influenced by differences between trial types rather than running the RSA on the premise pairs themselves. Furthermore, the RSA now does not include stimuli at extreme ends of the transitive hierarchies (i.e., A and G in either the recent or the remote conditions), since they were only presented in the premise discriminations. Finally, the mixed-effect models that implement each RSA now include an additional predictor of no-interest that accounts for pattern similarities attributable to the temporal proximity of trials and the decomposition procedure. For more details, please see our last response to the comments from Reviewer 1.

2. The modeling work could be more clearly explained, and more clearly connected to the other analyses. The behavior of the perceptron model was compared to human behavior, but the link between model representations and neural representations could be fleshed out more clearly by running a similar analysis on the model representations as on the neural data. The relationship between the inference model and the behavior is also somewhat unclear, as the fitting procedure is difficult to understand based on the methods provided. Also, best-fitting parameter values and fitted performance are not reported, making it harder for the reader to evaluate how reasonable the models are. It is also unclear whether the OR model corresponds to a reasonable theory of encoding-based inference, as the mechanisms involved are not specified in detail. For example, for a B vs. E inference test, the OR model assumes that any of the B>C, C>D, or D>E pair memories may be retrieved. How would the C>D memory be retrieved in this case, given cue items of B and E? Finally, a reasonable alternative model, wherein each item is associated with a value within the hierarchy, is not considered. In the case of each item being associated with a value, the improved performance for more distant pairs would be related to the larger difference in value, rather than a larger number of individual memories that could be retrieved as in the OR model.

   To address the concerns raised in the public review, explain the fitting procedure for the inference models in more detail, and report the fitted parameters and fitted model performance. Were there two separate fits, one to determine rp based on the premise performance, and another fit to inference performance using the fitted rp? Or was there just one fit? Were parameters estimated for each participant and/or condition separately? On page 27, why were sets of Ri were repeatedly sampled over 1000 iterations? Isn't the likelihood function computable here, without having to do repeated sampling? For the inference models, consider the possibility that inference may be supported by retrieval of the value associated with each item. Such a model might have somewhat different predictions from the OR model. Even if the predictions would be the same, a discussion of different potential underlying mechanisms would still be useful for the paper.

We thank the reviewer for these useful suggestions. As recommended, we have now included further details regarding the theoretical motivation for the inference (AND/OR) models and how their free parameters were estimated (see page 12 and pages 26-29). To directly answer the questions raised, r_p values only relate to premise discriminations as they represent the estimated probability of retrieval for each pre-trained pair. Random samples of these values were drawn from their likelihood distributions (one round of sampling for each inferred discrimination) and these samples were then used to compute the distribution of g_i values (the probability of successful inference) under the AND and OR models (at the same time). This random sampling was necessary as the distribution of g_i values is difficult to express directly since it jointly depends on the likelihood of all r_p values relevant to the inference under consideration. The cross-entropy statistics plotted

in Figure 5 indicate goodness-of-fit statistics for each inference model and these were separately computed for each 2x2 (training schedule by session) condition. However, in light of the reviewer's comments, we now include rank correlation statistics indicating the strength of association between actual and model-predicted performance across inferred discriminations (again split by condition, see Supplementary Table 4).

With regards to the MLP, we chose not to replicate the RSA applied to the imaging data since we do not expect that these models learn distributed structural representations that the RSA is suited to detect. Instead, we use this analysis to show that progressive training exposes some network architectures to an error gradient that facilitates the learning a generalised value for each stimulus. The previous version of our manuscript failed to fully discuss this result and only included a figure illustrating this effect in the supplementary results. We have now address this by including a fuller discussion of the MLP findings (extract copied below from page 8), and moved the aforementioned figure into the main text (Figure 2B). We thank the reviewer for highlighting this issue and prompting us to revise the manuscript as we have.

> *The above demonstrates that progressive training can yield better inference in some MLPs, particularly those with only a single hidden neuron. To explore the reasons for this, we computed the linear activation of each output neuron (prior to SoftMax normalisation) that resulted from stimulating the corresponding input neuron with a one-hot vector. We denote this activation, $\Delta\alpha_i^3 \mid \alpha_i^1 = 1$, for the ith input/output neuron, where superscripts denote layer indices. Figure 3B plots these values as a function of training trial, averaged across runs for MLPs with a single hidden neuron. This showed that activity in the output corresponding to stimulus 'A' is generally higher than the activity of all others, activity in 'B' is next highest, and so on. As such, progressive training produces activations in each output neuron that faithfully represents a generalised value for the corresponding stimulus as long as that stimulus is presented on the input layer. We note that this is also evident in MLPs with more than one hidden neuron yet, the value representation is much weaker, see Supplementary Figure 1.*
>
> *This generalised value gradient develops because learning the first discrimination ('A>B') tunes the MLP to increase the activation of the 'A' output above all others. When the next discrimination is introduced ('B>C'), activity in the 'B' output starts to increase yet, because the 'A>B' discrimination is still being tested, activation in the 'A' output also must rise to maintain performance. This pattern continues down the transitive hierarchy as long as older discriminations are presented often enough to avoid catastrophic interference. As such, progressive training can yield a very specific error gradient that allows the network to learn a generalised value for each stimulus. The same is not true for MLPs trained via an interleaved schedule. Here, activity levels in output neurons are approximately uniform for all stimuli that are rewarded during training (i.e., excluding stimulus 'G'). Instead, networks trained in this way tended to learn the discriminations by inhibiting the activity of non-target outputs in response to the presentation of specific premise pairs.*

Finally, the reviewer suggests that it may be beneficial to explicitly test a value-based generalisation model alongside the AND and OR models. Alternately, the reviewer suggests that we discuss the relationship between value-based models, and the OR model if they make similar/identical predictions. Indeed, we can think several value-based mechanisms that would produce identical

predictions to the OR model and so have now amended the manuscript to mention this (see above extract from page 8). We are also aware of one value-based model that may make slightly different predictions to the OR model (e.g., Ciranka et al., 2022). Unfortunately, however, when taking this modelling approach, it is not possible to estimate finite relative values between stimuli if performance is at or near ceiling (as is the case in our study). Furthermore, this approach would require us to make strong assumptions regarding the relationship between unobserved learning parameters and behavioural performance that would be very different from those underlying the AND and OR models. We believe that a core strength of our modelling work is that both models rely on very similar assumptions, are based on exactly the same performance data, and are general enough to represent a wide range of theoretical positions.

3. The relation between the present findings and previous findings is somewhat oversimplified. In particular, potential differences between transitive inference, in which participants learn an item hierarchy, and associative inference, in which participants infer associations between indirectly related items, are not considered. Transitive inference may more easily rely on encoding mechanisms, as the overall structure is much simpler. Also, while the study finds evidence that progressive learning helps to support transitive inference, the conclusions in the discussion are overly broad, saying for example that "We speculate that progressive exposure may facilitate inference whenever generalisations must be made on the basis of structural representations of the information." Here, that has only been shown to be the case for hierarchical structure. Previously, interleaved learning has been shown to facilitate generalization when participants learn to categorize items.

We thank the reviewer for highlighting that we had not fully discussed the similarities and differences between our results and those reported in previous studies. We have now added a more thorough discussion of the previous literature including how our design and findings differ to those of previous studies (see pages 2-3, and 18). We have also clarified our speculation that progressive exposure may sometimes facilitate generalisation. Specifically, we emphasise that this only relates to generalisations that depend on structural representations, and we highlight that this may be a reason why some forms of generalisation benefit from progressive exposure whereas other do not.

4. The analyses approach transitive inference as depending either on encoding or on retrieval, but do not consider possible combinations of these mechanisms. For example, retrieval-based inference might be recruited when encoding-based inference fails. The relatively similar fits of the AND and OR models in the interleaved condition may reflect a greater reliance on retrieval, even though the OR model provides a better fit overall.

We appreciate the suggestion that encoding- and retrieval-based mechanisms may be evoked following a single inferential cue, either in series (if one mechanism fails), or in parallel (until a metacognitive response criterion is reached). However, we reason that on any one trial, each behavioural response is only influenced by one of these mechanisms alone. Since our a priori hypotheses explicitly stated that encoding-/retrieval-based mechanisms would be preferred in the progressive/interleaved conditions (respectively), we constructed the AND and OR models to test these trial-level predictions. More aggregate evidence in favour of one of model over the other was therefore sufficient to test our hypotheses, whereas evaluating the fits of hybrid models would not. It is for this reason that we do not consider hybrid accounts within the current manuscript.

5. The details of the functional scan are unclear. If there were 192 trials, with 6.5 s per trial and 16 null events of 6.5 s each, then the functional scan would take about 22.5 minutes to run. The manuscript does not mention any division of these trials into separate runs. If there was just one scanning run, then fatigue and motion would be a substantial concern, especially given that no

exclusions of participants or samples based on motion are mentioned in the paper. Give more detail about how the trials were scanned. Were all the scanned trials within the same scanning run? Were there any exclusion criteria for removing scans with excessive motion?

We thank the reviewer for raising these points and sincerely apologise for the omissions. The methods section has now been updated to include all the requested details. In our original submission we had neglected to note the number of participant exclusions and reason for them (extract taken from page 23):

> *Data from 5 participants could not be included in the final sample because of problems with fMRI data acquisition (one participant), excess of motion-related artifacts in the imaging data (three participants), and a failure to respond during the in-scanner task (one participant). After these exclusions, the final sample included 34 participants (16 females) with a mean age of 25.9 years (SD = 4.60 years).*

6. Any possible effects of response time on univariate activation during the scanning phase are not considered. If response time is slower for some trials, that could lead to longer activation in a brain region involved in making that response; this could cause, for example, greater activation on inference trials compared to premise trials. This is not a major concern, as the reported effects are generally contrary to what would be expected based on that confound, but the paper should discuss possible effects of response time nevertheless.

We thank the reviewer for highlighting these important considerations. Our general approach is to first test for BOLD differences between conditions without controlling for differential response times. Then, if this reveals notable effects, we run a set of stricter control analysis that attempt to regress out response times across trials. This approach affords us the maximum levels of statistical sensitivity in detecting BOLD effects related to task processing whilst the stricter control analysis allows us to interrogate whether effects can be ascribed to differences in processing time alone, or likely reflect fundamental changes in cognitive function. However, it not always possible to make this distinction since processing time and regional differences in BOLD activity may be closely correlated due to extraneous factors.

Contrary to the predictions of retrieval-based models, we did not observe BOLD activations that were uniquely associated with generalisation performance. Instead, BOLD effects in the right hippocampus and superior MPFC were positively correlated with performance on both premise and inferred trials. As the reviewer notes, these effects are in the opposite direction to those that would be expected under the assumption that they directly correspond to processing time. Furthermore, since they were not central in testing our a priori hypotheses, we have not run any follow-up analyses controlling for response times.

7. Analyses examining structure within each hierarchy and common structure among hierarchies provide two useful measures of neural representation, but another important aspect to consider is the differences between the hierarchies. Do hippocampus, entorhinal cortex, and medial prefrontal cortex represent items from the same hierarchy as more similar compared to items from different hierarchies?

We appreciate this suggestion but have not run this test since we are no longer sensitive to across-hierarchy distance effects that suggest a common neural code across stimuli trained on each day. As such, the suggested analysis would be of little value, especially since they are not central to our a priori hypotheses.

8. To help connect the perceptron model to the neural analysis, it might be helpful to show how similarity on the hidden layer or the output layer varies with distance between items. This would allow assessing qualitative predictions; for example, does representational similarity in the model follow a linear function of distance in the hierarchy, as observed in the neural data?

We thank the reviewer for raising this point. As noted above, our previous submission did include an analysis illustrating how progressive training fosters a generalised value representation on the output layer which can drive distance effects. However, we failed to properly discuss these findings and only included a relevant figure in the supplementary figures section. In light of the reviewer's suggestion, we now centrally discuss these results in the main results section.

Interestingly, we find that the value representations gained by MLPs after progressive training are not linear but logarithmic (similar to those identified by Ciranka et al., 2022; although the underlying reason for this similarity is attributable to different causes - being driven by interference effects in the MLPs, and an asymmetric learning rule in Ciranka et al.). Furthermore, we do not claim that the distance effects observed in the hippocampus and MPFC are strictly linear. Instead, we simply use a linear-distance metric as a convenient way to model the predicted effects in a way that should be sensitive to most of the signal. Indeed, there are good reasons to believe that hierarchical distance representations in the brain are not linear; the successor representation model tends to produce exponential distance effects (see Supplementary Figure 4), and Ciranka et al. found good evidence that learners tend to acquire logarithmically spaced distances.

Minor recommendations

9. "The entorhinal masks were derived from the maximum probability tissue labels provided by Neuromorphometrics Inc." - What methods were used to determine those masks? Also, how exactly were the MPFC ROIs defined? Which clusters were included in each ROI?

The Neuromorphometrics probabilistic atlas is widely use in fMRI studies. Based on the data from 114 participants, the methods used for volume segmentation and parcellation are described in great detail by the vendor (see http://neuromorphometrics.com/Seg/).

10. "The models also included a fixed-effect predictor of no-interest that accounted for trivial correlations between the wall-texture representations associated with the task structure (see 'MRI pre-processing')." - How was this confound regressor was generated? Please give more detail. What is meant by "trivial" here?

We thank the reviewer for highlighting this lack of clarity and have now amended the methods section to include more details of this nuisance regressor (see extract from page 30 copied above in response to point 3 from Reviewer 1).

11. "Finally, the LMMs included an extensive set of random intercepts and slopes that accounted for all dependencies across wall-textures and participants." - This is not clear. The parameters should all be explained clearly.

It would be unfeasible for us to list all the random effects parameters from each LMM. For this reason, we simply state that random intercepts and slopes were generated to account for all dependences related to wall-texture stimuli (i.e., item effects), and participants (i.e., subject effects). We openly provide the analysis scripts for these models on the OSF so that readers can interrogate specifics and replicate the analyses. However, we had not directly linked to these scripts within the manuscript and so have now included URLs at relevant locations (see pages 31 & 32).

Reviewer 3

This paper present works showing 1) a neural network and humans generalise better in transitive inference tasks when item pairings are shown progressively versus interleaved. 2) That human behaviour on unseen pairings is better explained by a model that retrieves a constant number of memories versus a model that needs to retrieve all memories to 'chain' together item pairings. The authors suggest this is evidence for a generalised metric space encoding/mapping the transitive inference task. 3) Pattern similarity analysis on fMRI data is consistent with patterns representing 'location' in the transitive inference space in MTL and MPFC, further evidence for an encoding/mapping of the task. 4) MPFC codes two different transitive inference tasks similarly, suggesting that hierarchy of abstraction from MTL to MPFC.

Overall, I think the fMRI results are interesting. I am unsure, however, how to interpret the neural network results (see below).

Once again, thank the reviewer for their very constructive comments a hope that our amendments and accompanying responses address all their concerns and outstanding questions.

1. The single linear hidden neuron: This makes it impossible to perform well on the cross-entropy loss. The best you can do is to have the weights w_i_2 decrease from i=1 to i=7, though this is still not very good - i.e., the network will predict A even when A is not presented. It renders the input weights (w_i_1) pointless - they can only affect the 'slope' of the softmax. You would likely get the same results would appear without any input nodes and just the single node (always with activity 1) projecting to 7 output nodes. "Nonetheless, solutions that do yield near-perfect performance on both premise and inferred discriminations do exist." This is not possible for the reasons discussed. It is possible when using the 2-alternative choice testing metric post-hoc, but it is not mentioned at this point that this is what you do.

We thank the reviewer for these comments but remain confident that MLPs with a single hidden neuron are able to perform well on both premise and inference trials, even when tested using the cross-entropy cost function rather than the 2AFC metric. Critically, this ability depends on weights and biases between both input and output layers that can exploit quadratic non-linearities to activate specific output units. For instance, in the scripted example that we provide on the OSF, the sum of SoftMax outputs for non-targets is *p* = 0.011 on premise trials, and *p* = 0.005 on inference trials.

Our reason for including the 2AFC metric was that we believed it constituted a stricter test of performance since it directly compares the target and non-target outputs that directly correspond to the input stimuli (which tends to be highly activated on any given trial). However, we do agree that it was an oversight to exclude cross-entropy performance measures in the original manuscript and so have now provided figures and tables of both metrics. Moreover, in light of the comments from both reviewers 1 and 3, our analysis now tests the effects of each training condition in MLPs with more than one hidden unit. This demonstrates that, while premise performance does increase with more hidden units, the gains are relatively modest.

2. The hyper-parameters A learning rate of 0.1 is high. In particular, the interleaved case is going to struggle with this since it receives large and seemingly inconsistent weight updates. I suspect that with a lower learning rate (and more training data) the interleaved model would work better. Choice of 10 x training data of human participants. Again, it feels as if you're in a goldilocks regime. Afterall, it is known that such progressive training (in the extreme case) leads to catastrophic forgetting.

We entirely agree that a learning rate of 0.1 is relatively high and so thank the reviewer for raising this point. As such, have now replicated all the MLP experiments (including the newly added analyses) with a more typical learning rate of 0.01 which produced an identical pattern of results (see extract below from page 25). Furthermore, we now note that the original learning rate was chosen to allow sufficient levels of learning with 10x the training data given to human participants.

> *Backpropagation was then used to adjust all network weights and biases with a learning rate of 0.1. This rate parameter was chosen to allow for sufficient levels of learning following 3,600 training trials (10 times the number used in the pre-scanner task, see https://osf.io/uzyb7/). However, to examine the sensitivity of our results to this rate, we ran a supplementary analysis that involved training the MLPs with a learning rate of 0.01 over 36,000 trials. This yielded an identical pattern of results to those presented above (see Supplementary Results).*

3. "The goal of this analysis was to characterise general effects of each training procedure on learning and inference." If you want to characterise the general effects then you'd need to show this is simulations with more than these specific network/hyper-parameter choices, and it should be a network that can actually solve the task!

As noted, we do believe that all of the MLPs included in the analysis are able to acquire high levels of performance on both premise and inferred discriminations. Furthermore, we have now extended our analysis to test the effects of progressive vs interleaved training in MLPs with different architectures and hyperparameters.

4. Overall, it seems that the results are specific to the chosen network and hyper-parameters. That's fine if the aim is to simply say there exists a network where this progressive training scheme leads to better generalisation. But in that case, since the model is not one that informs us of computation, it's hard to know what to take from it. If the aim, however, was to show something more general about progressive versus interleaved training, then there needs to be a proper characterisation i.e., not just looking at one set of hyperparameters.

We thank the reviewer for these comments as they have prompted us to review the general claims that we were making in regard to progressive vs interleaved training schedules. While we have extended our analyses to examine different architectures and hyperparameters, we recognise that the relative effects of progressive training are still relatively specific to certain networks and particular problems. As such, we now emphasise this point in the manuscript, and discuss why progressive training may have particular effects in certain networks (see page 19-20):

> *Our behavioural analyses clearly demonstrate that progressive training substantially increases generalisation performance compared with randomly interleaving contingencies. This happens despite comparable accuracy in remembering the directly trained (premise) contingencies that generalisations were based upon. We observed similar effects in an MLP that was specifically structured to allow the learning of low-dimensional, compressed representations of the transitive hierarchy. Here, progressive training in MLPs with a single hidden neuron boosted successful inference. This happens because progressively ordering task contingences induces an error gradient that favours learning the relative value of all stimuli (A>B>C…; see Results). Progressive training did not, however, confer the same advantage for MLPs with two or more hidden neurons since these networks were biased toward learning localist representations of the premise pairs that did not encode the higher-order transitive structure.*

Using similar MLPs, Flesch, Juechems, Dumbalska, Saxe and Summerfield (2021) found that networks initialised with high-variance parameters tended to learn 'lazy' representations of stimuli in a categorisation task that indiscriminately preserved irrelevant features. On the other hand, when the networks were initialised with low-variance parameters, they were biased towards learning low-dimensional (compressed) representations of the input space that linearly encoded task-relevant features. These highly structured, 'rich' representations were learnt slower than lazy ones but were more resilient noise in the input layer. Our findings extend these results by highlighting that progressive training may selectively promote generalisation when rich coding schemes are acquired during training.

5. AND/OR model: The crux of these models is that the AND model requires all chains to be retrieved, whereas the OR model only requires one (or a constant number) of the elements in the chain to be retrieved. This means the OR model works better with longer distances between items since the odds are higher that a randomly retrieved memory will be in the chain, while the AND model performs worse since it needs to retrieve more memories. While I agree that one means of differentiating 'encoding' vs 'retrieval' models is on how generalisation performance changes with transitive distance, another one is how computation time changes with transitive distance. In this case 'retrieval' models predict computation time scales with transitive distance since the whole chain must be retrieved, while encoding models have constant computation time - just retrieve the encoding of each item and compute the vector between them. Your reaction time data (from figure 3) looks like it would again support a constant time 'encoding' model à something that you could verify if you wish.

We thank reviewer for this very good suggestion. We had considered extending the AND and OR models to test them against the response time data. Indeed, we believe that such extensions will contribute additional evidence to support our claims. However, we have been unable to extend the models in this way for one key reason. We assume that response times on inference trials are influenced by two distinct factors: *1)* a visual encoding processes associated with recognising each individual wall texture, and *2)* a memory retrieval process that depends on transitive distance. Unfortunately, despite our best efforts, we have been unable to satisfactorily estimate the unique effects of each factor without relying on specific assumptions that could bias our results. As such, we have made the decision to restrict the AND and OR models to the accuracy data alone.

6. fMRI: As mentioned, I am not an expert in fMRI, and so cannot comment on the technical details of the analyses. Nevertheless, I found the results here intriguing. In particular the results that MTL encodes 'environment' specific maps, while mPFC a map common to both 'environments'. While this is, to my knowledge, the first evidence of this in transitive (/sequential) structures in humans, there is some evidence to suggest a similar coding mechanism in rodents (Zhou et al., 2020, Nature; Kaeffer et al., 2020, Neuron) which would be worth referring to.

We thank the reviewer for these comments and have now referred this rodent literature in the introduction section.
