# Supplementary material for: Hippocampal and medial prefrontal cortices encode structural task representations following progressive and interleaved training schedules
Source: PLoS Comput Biol. 2022 Oct 17;18(10):e1010566. doi: 10.1371/journal.pcbi.1010566 (PMC9612823; doi:10.1371/journal.pcbi.1010566)
Supplement: Attachment — Submitted filename: Berens-Bird_2022_ResponseToReviewers_R04.pdf [file pcbi.1010566.s009.pdf]

We thank the reviewers who provided feedback on this manuscript, all of which was extremely constructive. We have endeavoured to address each point that was raised as fully as possible and feel that the amended manuscript is much improved in quality. Below we have copied each of the reviewer comments along with are our responses (in bold), and quoted sections of manuscript (indented).

Reviewer 1

This paper examines the effects of training schedule (progressive vs. interleaved) on the representations supporting transitive inference, using behavioral modeling, computational simulation, and fMRI. The authors hypothesize that interleaved training (pseudo-random presentation of premises) will lead to retrieval-based inference, such that individual premises are retrieved and integrated at the time of test; while progressive training (where each premise is well-learned before introducing another) will lead to the encoding of unified representations of the hierarchy during training. They also examine whether these different strategies are more or less prominent after short vs. long delays, hypothesizing that consolidation will increase the reliance on unified, "map-like" representations. The results showed clear advantages for progressive training over interleaved training in all conditions. Consistent positive symbolic distance effects (transitive slopes here) suggest a reliance on encoding-based inference rather than retrieval-based inference in both training conditions and both delays. The fMRI analysis generally supported the use of structural representations of the hierarchy rather than retrieval-based inference, though these effects were only apparent for more recently learned relationships.

The paper is generally well-written and clear. The theoretical background is mostly sufficient (with some important exceptions noted below) and the statistical analyses appear to be appropriate and well-documented. The fMRI methods appear to be sound, although since this is not my area of expertise my review focuses on the behavioral results and computational modeling. There are some notable strengths of the paper in terms of its originality and significance:

a) I agree that while both encoding- and retrieval-based mechanisms have been proposed as accounts of TI, there's a poor understanding of when people might rely on one or the other. So I found the overall research question to be interesting and worthwhile.

b) There are several existing computational models of encoding-based mechanisms for TI which can all capture the basic behavioral phenomena (like a positive symbolic distance effect). There's potential for model-based neuroimaging to make progress toward identifying which representations underlie behavior, particularly the kind of RSA approach used here.

c) Although both interleaved and progressive training have been commonly used in previous studies of TI, there are surprisingly few direct comparisons of different training schedules (particularly among humans). Aside from Markant (2020, 2021), the closest example would be Lazareva et al. (2020), who compared training conditions that were similar to the present paper in a sample of rhesus monkeys. They also found support for a unified representation regardless of training condition (or to be more precise, they didn't find evidence that simpler associative models could account for performance in either condition). The authors may want to incorporate this study in their background.

d) The authors have provided what appears to be a comprehensive repository of their code and materials on OSF.

I had some (relatively minor) concerns about the coverage of existing work on theories/models of TI (see point 1 below), and limitations with the task design which the authors may want to address in their discussion (see point 2).

I found the computational modeling components (both the MLP and the AND/OR comparison) to be the most problematic (see points 3 and 4 below). While both sections seem to be technically sound, they seemed to stand apart from the rest of the paper (particularly the MLP results, which seems to be an interesting modeling exercise but without any clear implications for how people perform TI). I expand on these points below.

We thank the reviewer for their very constructive and positive comments. We address their specific comments point-by-point in the section below. We have now incorporated the Lazareva et al. (2020) into the introduction (see extract from page 2 copied below).

Main points

1. In some ways the paper does not sufficiently engage with the existing (large) literature on TI. Although I agree on the importance of the overall question of when/whether encoding- or retrieval-based mechanisms drive TI, the existing literature already suggests that encoding-based mechanisms dominate much of the time (indicated by positive transitive slopes seen in many studies). Besides the Banino et al. (2016) paper (which involved an associative inference task, not TI), I'm not aware of other studies that have shown negative transitive slopes as expected from a retrieval-based account (although there might be some evidence that slopes are more negative for individuals who are "unaware" of the hierarchy by the end of training, see e.g. Ryan et al., 2009).

We address these comments together with the points raised in the next paragraph.

More importantly, positive transitive slopes could be consistent with a number of different "encoding-based" mechanisms/models, including forms of reinforcement learning (e.g., Ciranka et al., 2021) and more map-like representations (like the particle filter model in Kumaran et al., 2016), and some other simpler associative learning models (see Vasconcelos, 2008). It's not altogether clear which of these are consistent with the "structural codes" described by the authors, or if it's possible to distinguish between these mechanisms on the basis of the present results. The authors discuss a couple of more recent proposals (the TEM and SR), but these other existing accounts are largely missing from the discussion.

We agree with the reviewer that the majority of previous studies report positive transitive slopes, which is suggestive of encoding-based mechanisms. We also acknowledge that there are more encoding-based accounts than we covered in the manuscript. We have now included additional references (throughout) and text (copied below) to discuss these points, alongside a fuller justification of our hypotheses.

Page 2:

> *Retrieval-based models predict that generalisation performance decreases when inferences require integrating information over more independent memory traces (so-called, negative transitive slopes; see [3]). However, encoding-based models often predict the opposite relationship, e.g., generalisations are easier when comparing stimuli that are separated by larger distances in an inferred hierarchy (positive transitive slopes). This is because the information required to discriminate stimuli based on their relative positions in an abstract task space becomes increasing salient with larger distances [22]. In support of retrieval-based mechanisms, there is clear evidence of negative transitive slopes when inferences involving distinct episodic memories [5]. Nonetheless, most studies have reported positive transitive slopes consistent with encoding-based mechanisms, particularly in transitive inference paradigms [9,23–26].*

Aside from retrieval- and encoding-based models, it has been suggested that above chance performance on transitive inference tasks can result from stimulus-reward associative learning simply because, during some training procedures, stimuli at the top of the hierarchy tend to be selected more often and so are more commonly associated with reward [27–30]. However, more recent research has shown that these simple associative mechanisms are unable to account for all transitive inference behaviours [9,23,25,26,31–35]. Moreover, participants in the current study were able to perform inferences despite a receiving a pattern of reinforcement that was entirely incompatible with stimulus-reward association learning (see https://osf.io/cteg9). As such, we do not consider these models any further in the current study.

Page 4:

As mentioned, many studies have rereported positive transitive slopes that are indicative of encoding-based generalisations. However, it is noteworthy that most of these studies have either employed progressive training schedules [23,26], or provided explicit feedback to inferred discriminations which can confound distance effects with differences in how often stimuli are rewarded [22,24,25]. Here, we directly tested the predictions of retrieval-/encoding-based generalisation mechanisms following both interleaved and progressive training schedules, in a feedback-free inference test.

As the reviewer notes, the main purpose of the study was to test whether the reliance on encoding- or retrieval-based mechanisms was influenced by the training schedule used, and/or the retention interval between study and test. We justify these hypotheses by highlighting that: *1)* interleaved training has been found to promote hippocampal pattern separation (Favila et al., 2016), whereas blocked training promotes pattern completion (Schlichting, et al., 2015), *2)* interleaved training may emphasise difference between studied items/episodes, whereas blocked training may emphasise their commonalities (Carvalho & Goldstone, 2014), *3)* progressively training neural networks results in representations that directly express generalised stimulus information consistent with encoding-based models (Flesch et al., 2018; our MLP analyses), and *4)* offline hippocampal replay during sleep is thought to promote the learning of generalised representations that support encoding-based inferences (Lewis & Durrant, 2011; Liu et al., 2019).

While we certainly feel that our results shed light on how encoding-based learning is instantiated in the brain, we did not design the study to adjudicate between competing encoding-based models (though we are keen to pursue this in future work). To clarify this, we have now included further discussion of how our behavioural and fMRI results relate to different encoding-based models:

Page 5:

In contrast, a representational similarity analysis (RSA) supported encoding-based models. Specifically, consistent with multiple encoding-based accounts, we identified RSA effects in the hippocampus and MPFC suggestive of structural representations that expressed inferred relationships across the whole transitive hierarchy.

As a side note, I was interested in the discussion of the successor representation as a potential alternative model (pg. 44). There seems to be some simulation work in the supplementary materials but this is only briefly mentioned in the discussion--it's unclear why this doesn't figure more prominently in the paper (there are also no corresponding methods to explain how they obtained the results in that figure).

The point we wanted to make was that successor representations are able to represent a chain of items if those items are experienced co-occurring in adjacent pairs. However, this representation would not include any information about which items are rewarded over others – this depends on an additional mechanism.

The original inclusion of the supplementary figure about successor representations was to provide some examples as to how successor representations might be used to represent a transitive hierarchy and when (and why) this might be advantageous. However, this relates to all transitive inference tasks in general rather than being specific to our study. It's clear that the original figure and its legend were not easily interpreted by both reviewers. Rather than devote more space to explaining this, we tend to agree with Reviewer 2 that its inclusion represents a "departure from the current study". We have therefore decided to remove it.

2. There are some features of the task design which may have contributed to the reliance on an encoding-based strategy. The authors may want to consider how these limited their ability to detect retrieval-based inference and the generalizability of their results.

   First, in the interleaved training condition there are many cases in which overlapping premises were experienced in successive trials, like the "chained" sequences in Markant (2021) which were associated with improved inference and greater explicit awareness of the hierarchy (the example in Figure 1C shows that this happened a fair amount of the time in the present task). This seems to run counter to the idea that "interleaved learning highlights the differences between items" (pg. 3), since it draws attention to shared elements across overlapping pairs. A training sequence without any overlap between successive pairs might be more likely to produce retrieval-based inference in line with the authors' expectations.

This is a very interesting point. We feel that it is beyond the scope of the present study to run an additional experiment to test whether an interleaved training schedule that explicitly avoids chaining would be more likely to produce retrieval-based inferences. However, it is noteworthy that our interleaved training schedule presented participants is relatively few chained sequences. Specifically, out of the 360 training trails, there were an average of 75.12 'chains' with a length of two, 10.23 chains with a length of three, and a negligible number of chains (1.51) with a length of four or more discriminations (see figure copied below). We now discuss this on page 21:

> *For participants in the interleaved learning condition, all discriminations were presented in a pseudorandom order such that there was a uniform probability (1/6) of encountering any one discrimination on any particular trial (see Figure 1C). Given that 'chaining' overlapping trails in an ordered sequence (e.g., 'B>C' followed by 'C>D') may facilitate encoding-based generalisations [45,46], it is noteworthy that our interleaved training schedule presented participants is relatively few chained sequences. Specifically, out of 360 training trails, there were an average of 75.12 chains with a length of two, 10.23 chains with a length of three, and a negligible number of chains (1.51) with a length of four or more discriminations.*

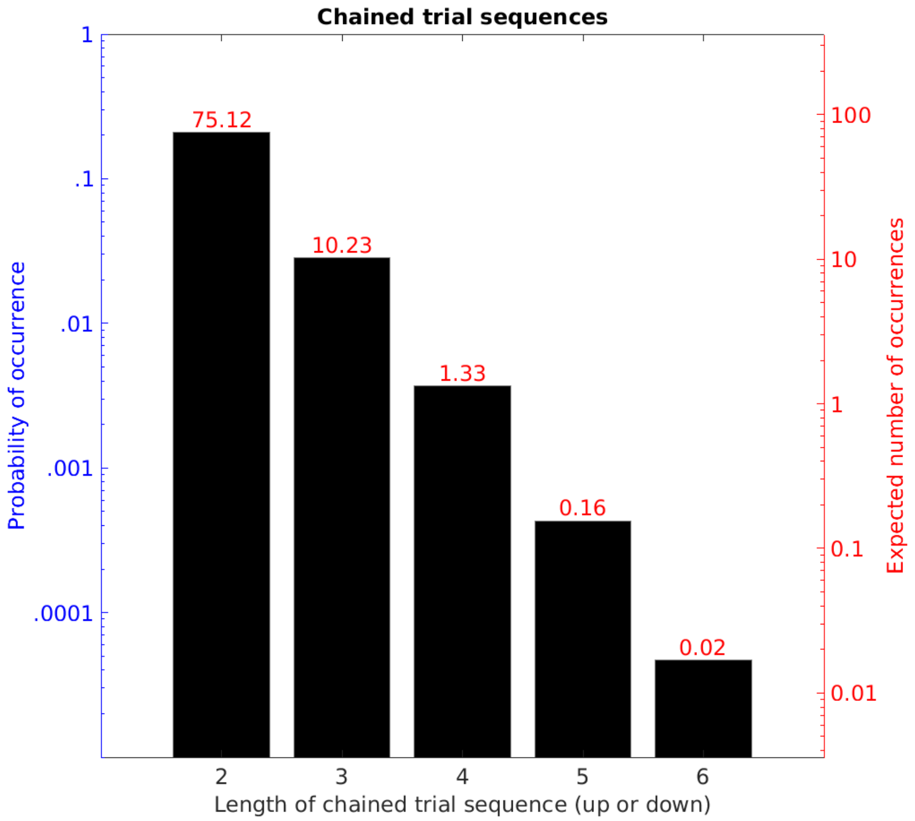

Second, when there are separable stimulus features people may also try to generalize based on those. The manuscript doesn't include many example of the textures, but looking at them on OSF there appear to be some dimensions that varied across items (e.g., color, size of the bricks). If people are given vague instructions about the task (i.e., to learn how the texture is related to reinforcement) then it would be reasonable that they compare across premises as they try to find a stimulus cue that predicts reinforcement. Granted that kind of strategy would not be successful in the task, but it's another factor that would lead to comparison across premises or attempts to form a more abstract representation of the hierarchy.

This is an important point and so we have updated the methods section to be clearer about the instructions given to participants. Participants were told that each wall texture should be considered as a single separate 'pattern', and that may either conceal the reward or not, depending on the other wall texture presented within the scene (see extract from page 22 copied below). We therefore consider it unlikely that participants would seek to identify individual elements or dimensions across multiple items that could predict the outcomes.

> *Regardless of the training condition that participants were assigned to, all pairwise discriminations were tested 60 times each by the end of the training procedure (i.e., 360 trials in total, ~37 minutes). Before the first training session, participants were briefed on the experimental procedure and told that the wall textures were the only features that predicted reward. These instructions specified that each wall texture should be considered as a single separate 'pattern', and that may either conceal the reward or not, depending on the other wall texture presented within the scene. They were not given any other details regarding the number or type of discriminations.*

3. I struggled to understand the rationale for the comparison involving the AND and OR models (pg. 12). The AND model is a sensible (if simplistic) model of retrieval-based inference (a similar

approach is taken in Ciranka et al., 2022). But I don't see the logic for the OR model, such that retrieval of a single premise leads to reactivation of the full representation of the hierarchy (or at least, the portion necessary to perform the inference at hand).

We appreciate that our previous description of the OR model did not properly motivate its structure and so we thank the reviewer for raising this issue. The core assumption underlying the OR model is that, as a result of training, participants can access a unified structural representation describing the '*associative distances*' between all stimuli (i.e., 'A-B-C-D-E-F-G', implies distances of: [A,B]=1, [A,C]=2, [B,E]=3). This assumption is shared by algorithmic-level models of encoding-based generalisation (e.g., Whittington et al., 2020; Momennejad, 2020; Stachenfeld et al., 2017). Nonetheless, in order to make a successful inference, knowledge of these associative distances must be evaluated alongside the reward contingencies enforced during training (e.g., 'B>C' and/or 'C>D', where '>' indicates the preferred stimulus).

Previously, we had specified the OR model in a way that allowed the recall of *any* mediating contingency to facilitate successful inference (for example, to evaluate B?F, it would have been sufficient to retrieve 'D>E'). However, in light of the reviewers' comments, we acknowledge that this structure is difficult to justify as when faced with 'B' and 'F', it is unlikely that the premise D>E would be retrieved. As such, we have now restricted the OR model to presume that successful inference is only possible following the recall of either contingency directly related to the on-screen stimuli (e.g., inferring 'B>F' depends on the recall of 'B>C' or 'E>F' alone). We now discuss this updated model structure and clarify the underlying assumptions within the results (page 9, extract copied below), and the methods (pages 24-26):

> *In contrast, the encoding-based OR model assumes that participants can access a unified structural representation describing the associative distances between all stimuli. Nonetheless, in order to make a successful inference, knowledge of this associative structure must be evaluated alongside the reward contingencies indicating which of the presented stimuli is higher in the reward hierarchy. When making an inference (e.g., B>E), it is therefore sufficient to recall only one of the contingencies indicating which stimulus should be preferred (e.g., B>C), or which stimulus should be avoided (e.g., D>E).*

The authors state that these are not meant to be process models, but that is at odds with some other text that suggest mechanistic modeling, e.g.: "Our analyses show that the behavioural data are better accounted for by encoding-based generalisation mechanisms when directly compared with retrieval-based mechanisms" (pg. 4) (to me the heading "computational models of human inference" further gives the impression of process models of some type).

We apologise for the lack of clarity here. Using the AND & OR models, we aim to provide high-level descriptions of the relationship between premise-trial and inferential performance that are broadly consistent with retrieval- and encoding-based mechanism. To do this, both models attempt to predict inferential accuracy by assuming core tenants of retrieval- and encoding-based mechanisms, and by relying on as few free parameters as possible (only one parameter is fitted to the inference data in each case). As such, while the AND & OR models do rely on some assumptions regarding the representations involved in transitive inference, they do not directly describe how these representations are implemented or manipulated. Given this, each models provides descriptions at the computational level-of-analysis (Marr, 1976). As the reviewer notes, the previous version of our manuscript used language that was not consistent with this modelling goal and so we have adjusted the main text in several place to address this (extracts copied below). Nonetheless, we remain

confident that comparing goodness-of-fit statistics across models tests their core assumptions and so can provide differential levels of support to encoding- and retrieval-based mechanisms.

Page 4:

> *Computational models that captured broad predictions of retrieval- and encoding-based models revealed that our behavioural data are better accounted for by encoding-based mechanisms.*

Page 24:

> *Note, these models are not intended to be process model that describe how humans solve the task at the algorithmic level. They are merely intended to describe the data and test whether the behaviour in each condition better accord with general predictions of encoding and retrieval models.*

It would also seem that these models are simply re-describing the "transitive slope" results. It is helpful to see the individual variability depicted in Figure 5, but does this add anything beyond showing the distributions of transitive slopes? If so I think the authors need to be more explicit about the contribution here, including how this might improve statistical sensitivity (as they suggested in a response to an earlier review). These shortcomings were especially notable since there are existing computational models of both retrieval- and encoding-based inference which might provide a stronger basis for detecting differences in strategies. In sum, I found this aspect of the modeling to ultimately be a distraction and to not add much beyond the behavioral results.

We thank the reviewer for their constructive suggestions here. The reviewer is correct that our computational models improve our statistical sensitivity. Moreover, they provide a more rigorous and direct test of our a priori hypotheses when compared to simply regressing transitive distance on accuracy or response time.

Firstly, the transitive slopes were not diagnostic of either encoding- or retrieval-based mechanisms in most experimental conditions. Although there was an overall trend relating distance and inference performance in the progressive learners (t_{804} = 2.48, p = .013), there was no such trend (either positive or negative) for the interleaved learners (t_{804} = 1.57, p = .116). Similarly, while there was an overall distance effect in the remote condition (t_{804} = 2.27, p = .023), this was not the case in the recent condition (t_{804} = 1.85, p = .065). Indeed, the corelations between distance and inferential accuracy that we did observe were largely driven by responses from progressive learnings in the remote condition (t_{804} = 3.07, p = .002). Furthermore, all trends relating distance to response time were non-significant (largest effect: t_{5301} = -1.62, p = .106). In contrast, our computational models allowed us to provide strong evidence for encoding-based mechanisms in all experimental conditions thereby falsifying our *a priori* hypotheses (interleaved: t_{128} = 2.69, p = .008; progressive: t_{128} = 8.18, p < .001; recent: t_{128} = 6.85, p < .001; remote: t_{128} = 7.48, p < .001).

Secondly, in contrast to examining the gradient of transitive slopes directly, our computational models account for differences in premise trial performance that may otherwise confound the analysis. For example, discriminations between more distant stimuli may be more likely to involve mediating contingencies that have been learnt either more or less well than most others, and this may have non-linear effects on inferential accuracy thereby obscuring, or even reversing, the expected transitive slopes. By explicitly modelling the profile of premise trial performance, our computational models predict the number of correct and incorrect responses for each type of inferential trial. We now explicitly make this point in the manuscript itself (see page 26):

Although the gradient of transitive slopes may also be used to differentiate encoding vs retrieval-based mechanisms [5,26], the approach outlined above explicitly accounts for differences in premise trial performance that can otherwise confound the analysis. For instance, discriminations between stimuli separated by a larger transitive distance are more likely to involve reward contingencies that can be remembered either better or worse than most others. This may have non-linear effects on inferential accuracy thereby obscuring, or even reversing, the direction of transitive slopes. Our computational models overcome this problem by explicitly accounting for the profile of premise trial performance.

Finally, as noted above, our updated OR model only estimates performance on each inference trial from two reward contingencies at a time (i.e., those related to the on-screen stimuli), regardless of the transitive distance involved. As such, the model no longer receives any information directly related to the transitive distance of each inferred discrimination. Despite this, it provides a better fit the inference data when compared to the AND model (t_{128} = 8.45, p < .001, as measured by the cross-entropy statistics), and accurately predicts the pattern of correct and incorrect responses within each individual (t_{31} = 7.82, p < .001, as measured by the Spearman rank correlation, ρ between predicted and observed inferential performance across trials, mean ρ = .484).

4. I found the MLP simulation results to be interesting and novel, but it was difficult to connect these results to the rest of the paper. The authors state that the models aren't intended to account for human performance or to adjudicate between encoding- and retrieval-based mechanisms, so it feels like quite a big detour in the middle of the paper with unclear implications.

While the MLPs analyses do produce interesting results and help to motive our a priori hypotheses, we agree that their inclusion does reflect a detour within the paper. As such, we have now move all the detailed MLP methods and results to a supplement (S1 Text), and only briefly discuss the findings of these analyses within the main text.

It was also unclear to me whether increasing the number of hidden units was meant to capture a transition from an encoding-based strategy to a retrieval-based strategy (akin to the approach taken by Banino et al., 2016, where both encoding and retrieval-based inference were examined in the same connectionist framework). This seems not to be the case here--e.g. supplementary Figure 1 shows that with 6 hidden units the network still learns a value gradient but with poorer discrimination between the items (so there should still be a positive transitive slope). So in the end the MLP captures the difference between interleaving and progressive training to some extent, but can't speak to the other factors in question (including differences between encoding/retrieval and recent/remote conditions).

Initially, our MLP analysis only included perceptrons with a single hidden unit. However, in a previous review, it was suggested that the effects of progressive training we identified may not generalise to MLPs with different architectures. As such, we extended our analyses to include MLPs with more than one hidden unit. This demonstrated that the beneficial effect of progressive on inference performance is indeed principally limited to MLPs with a single hidden unit. In other words, progressive training can significantly affect the type of representations that are learned by MLPs, but only when they are constrained to rely on low-dimensional solutions. This finding is consistent with those of Flesch et al (2021) as we discuss in the supplement:

Using similar MLPs, Flesch et al [1] found that networks initialised with high-variance parameters tended to learn 'lazy' representations of stimuli in a categorisation task that indiscriminately preserved irrelevant features. On the other hand, when the

networks were initialised with low-variance parameters, they were biased towards learning low-dimensional (compressed) representations of the input space that linearly encoded task-relevant features. These highly structured, 'rich' representations were learnt slower than lazy ones but were more resilient noise in the input layer. Our findings extend these results by highlighting that progressive training may selectively promote generalisation when representations using rich coding schemes are acquired during training.

The MLP results might be more impactful if a stronger case was made about the importance of the interleaved vs. progressive training comparison. Most of the paper is framed around the goal of using these different training sequences to expose different mechanisms for inference, but the training conditions themselves are not inherently that interesting. The authors do allude to the reliance on interleaving in machine learning in the discussion—perhaps there are broader implications for work in that area, but this would seem to be somewhat out of place in the present paper.

As noted, in light of the reviewers comments, we have decided to move most of the details about the MLP findings to a supplement rather than spend more time discussing the importance of the interleaved vs progressive training distinction.

Minor points

5. pg. 10: "Additionally, there was a significant 3-way interaction between training method, session and transitive distance, t(804) = 3.18, p = .002. This suggested that the effect of transitive distance was most consistent for remote discriminations in the progressive condition, t(804) = 3.07, p = .002, relative to all other conditions, t-values < 2.00 (see Figure 4B)." -- I don't follow this interpretation, what does it mean to be most "consistent"? From Figure 4B it looks like the effect of transitive distance is smallest in the progressive condition (and I'd be surprised if there was a difference between recent and remote for that condition).

We agree that our description of this 3-way interaction was ambiguous. In short, there was only a weak association between transitive distance and performance in the interleaved learners (t-values < 2). Nonetheless, in the progressive learners, inference performance significantly increased as a function of distance in the remote (t_{804} = 3.07, *p* = .002), but not the recent (t_{804} = 1.55, *p* = .122), condition. However, because our GLMM used a logit-link function, linear changes in these predictor variables did not correspond to linear changes in the probability of a correct response (p) and could also be expressed as lower variances in the probability estimates.

Since progressive learners tended to perform at ceiling in all experimental conditions, the significant association between distance and performance did not reflect a large change in the probability of a correct response (p; which only increased from 0.921 to 0.996). Instead, this increase principally manifested as lower variances in the probability estimates (see error bars in Figure 2B).

The reduced variance follows from the relationship between the expective value of the Bernoulli distribution ($E[p] = p$), and its standard deviation ($\sqrt{Var[p]} = \sqrt{p(1-p)}$), see the left panel of the below figure. Indeed, the rate of change of the standard deviation with respect to the estimated probability asymptotically approaches $-\infty$ as p approaches 1 (see right panel of below figure). This means that, small increases in probability estimates that are close to 1 are most visible as reduced variances in the observed probabilities.

We now clarify that the 3-way interaction does not correspond to a larger increase in the probability of a correct response within the progressive-remote condition, but mainly reflects performance becoming more consistently accurate with larger distances (see page 7-8):

> *Additionally, there was a significant 3-way interaction between training method, session and transitive distance, t(804) = 3.18, p = .002. This suggested that transitive distance most strongly correlated with performance in the progressive-remote condition, t(804) = 3.07, p = .002, relative to all other conditions, t-values < 2.00 (see Figure 4B). However, because performance in the progressive-remote is close to ceiling, this correlation does not correspond to larger increases in the probability of a correct response. Instead, it mainly reflects performance becoming more consistently accurate (i.e., lower variances in the binomial probability estimate) with larger distances.*

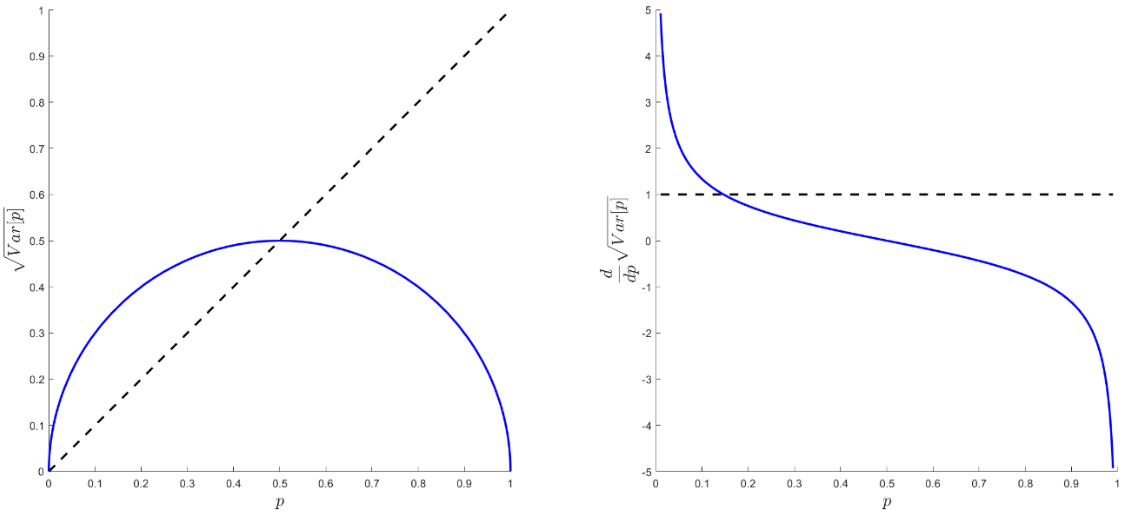

6. pg 11: For the GLM of RT, why is the DF so much larger than for the accuracy, when it is based on fewer observations?

When modelling the probability of a correct response, our unit of observation was the number of correct responses (k), to the $n = 8$ repeated test trials that presented the same discrimination. As such, the GLMM attempted to predict the number of correct responses with an unobserved probability estimate (p), i.e., $k \sim B(n, p)$. In this case, the DF relates to the number of unique discriminations multiplied by the number of participants. When modelling response times, our unit of observation was the response time on individual trials that were associated with a correct response. In this case, the DF relates to the number of correct trials by the number of participants.

Reviewer 2

Berens and Bird present a multifaceted study of transitive inference with a focus on different training schedules and consolidation. Through computational modelling, behaviour, and neuroimaging, they characterize how transitive inference is supported by interleaved and progressive learning schedules, hypothesizing that these schedules may encourage encoding- versus retrieval-based learning mechanisms. First, they find that simulations with MLPs show more consistent learning of inference relationships when trained with progressive schedules (notably, some interleaved schedules led to high inference performance but on average was at chance and highly variable). Second, they find strong a benefit in behaviour, notably for inference performance, for progressive schedules and demonstrate through a clever descriptive model analysis of premise pairs that participants were mostly likely engaged in encoding-based learning. Finally, the authors find that fMRI pattern similarity in hippocampus and mPFC is consistent with the transitive structure of the task. The authors argue that these findings collectively offer insight into how transitive inference learning can be optimized via progressive learning schedules and highlight potential neural mechanisms for building task-related representations although the link between behaviour and brain is murky.

The study is well conducted, the analyses are rigorous, and the interpretation of the findings is refreshingly honest. The comprehensive research approach that considers computation, behaviour, and the brain should be applauded and is a major strength of the current work. Also, the authors' dedication and actual implementation of open science practices is noteworthy. It is also clear that each step of analysis has been carefully considered (e.g., generating nuisance variables that represent confounds introduced by pattern estimation is very clever). Despite this enthusiasm, I have to hesitate on the overall contribution of this work. As detailed below, I have important questions about the central neural findings and the overall cohesion of this study's many components.

We thank the reviewer for this detailed and generally positive summary of our work.

Major comments

1. As noted above, I think the multifaceted approach of this study is a major strength. However, I feel that there is a notable lack of cohesion between the computational, behavioural, and neural components. For example, the MLP simulations (which were a surprise when first presented in the results section, more on that below) offer a computational starting point for considering the two training schedules and provide fairly straightforward predictions for human behaviour and neural measures, but how does this relate to encoding- vs. retrieval-based learning? And, the MLP findings seemingly had no conceptual bearing on the subsequent behavioural and neuroimaging analyses. This is largely a comment on framing and presentation; however, I think fixing this is necessary to fully evaluate the overall contribution of this work. A revised introduction that clearly walks through the overall approach and provides a motivation for the different components and how they are connected would greatly strengthen the manuscript.

The MLP analyses are indeed intended to "offer a computational starting point for considering the two training schedules" - particularly with respect to our prediction that progressive training facilitates encoding-based generalisations and improves inferential performance. However, these analyses did not directly relate to our subsequent computational models and neuroimaging analyses as the MLPs were not equipped with a retrieval-based generalisation mechanism. We therefore appreciate that the MLP analyses may be distracting to the general reader and so have moved them to a supplement (S1 Text).

2. The key finding, which is highlighted in the title, is that structured task representations are found in hippocampus and mPFC. This is mostly supported by the RSA findings showing a negative relationship between transitive distance and representational similarity. But, the link between

these neural signatures of task structure and transitive inference behaviour is weak. It is only those learners who perform poorly who drive this effect and it is stronger in the recent condition. The differences in how the mPFC and hippocampus effects relate to behaviour (e.g., that the hippocampus effect is strongest when behaviour did not suggest encoding-based learning) further clouds the interpretation. The result here is that the main finding seems to rely on a subset of the conditions tested and in a subset of participants who did not learn well. It is unclear what sort of neural representations supported successful learning. There may be some evidence that transitive structure is embedded in neural patterns, but it is not clear how well this is linked to behaviour (and in fact may be associated with poor learning or an unsuccessful strategy from those struggling with inferences). Given the clear evidence for a benefit in progressive training on inference performance, the neural findings based on a subset of participants in specific conditions feels like the two components are only loosely connected at best.

We disagree that our main findings rely on a subset of participants who did not learn well. Firstly, all participants were extensively trained on the premise pairs to the extent that their in-scanner performance was consistently close to ceiling, Pr(correct|Interleaved) = 0.925, Pr(correct|Progressive) = 0.916 (note that premise performance is numerically larger in the interleaved condition). Inference performance only represented half of the of in-scanner trials and was consistently above chance in all experimental conditions. This means that multivariate BOLD patterns were derived from trails which consistently yielded between 78.4% and 94.2% accuracy.

Secondly, while some pattern similarity effects were modulated by our two performance measures, the left hippocampus and left MPFC expressed structural representations that generalised across all participants in the sample. In the left hippocamps, we found an overall distance effect that does not depend on any subset of the data, t_{652} = 2.61, p = .009. While this just misses out on our strict threshold for statistical significance, 27 of the 34 participants exhibited a negative distance effect in this region which is significantly more than would be expected by chance alone (p < .001, binomial test). Furthermore, the mean Fisher-transformed correlation statistic (z = -0.136) is significantly less than zero when tested in a random effects analysis (t_{33} = -4.72, p < .001). We now report these supplementary results in the main text (see expert below from page 15). The left MPFC distance effect was indeed only evident in the recent condition, yet as training session was a repeated measures factor, the implied structural was present in the whole sample, t_{650} = 3.00, p = .003.

We appreciate that our results are nuanced by interactions that modulate the strength of structural representations. However, these interactions do not detract from the general trends noted above. In light of the reviewers' comments, we now express this more clearly within the main text (see extract copied below from page 15). Nonetheless, all effects cited in the main text directly relate to our a priori hypotheses and so it would be inappropriate to omit a subset of them in the interests of presenting a simplified picture. As to the relationship between inference performance and neural representations, it is notable that we explicitly falsified our a priori hypothesis concerning the effect of different training schedules. Consequently, our results are highly informative in that they provide convergent evidence that rules out various explanations for the differences in behavioural performance that we observed.

> *In the left hippocampus, we saw a main effect of distance that did not survive our correction for multiple comparisons; t(652) = 2.61, p = .009. While not reaching our strict criterion for statistical significance, 27 of the 34 participants exhibited the predicted distance effect in this region which is significantly more than would be expected by chance alone (p < .001, binomial test). As such, this effect is consistent with our prediction that representational similarity between wall textures should be inversely related to their hierarchical distance (see Figure 7B). The left hippocampus also produced a distance by transitive slope interaction, t(652) = 3.31, p = .001. Contrary to predictions, this indicated that distance effects were most strongly*

expressed in participants who had a relatively low (or negative) transitive slope (see Figure 9A). As such, the left hippocampus appears to encode structural task representations most strongly when behavioural performance is less typical of encoding-based generalisation.

In the left superior MPFC, transitive distance was negatively correlated with representational similarity in the recent, but not the remote, conditions; $t(650) = 3.59$, $p < .001$, and $t(650) = 0.113$, $p = .910$ (respectively). This resulted in a significant interaction between transitive distance and training session suggesting the presence of structural representations for recently learnt stimuli alone, $t(650) = 3.00$, $p = .003$ (see Figure 7C). On top of this effect, we saw a 3-way interaction between distance, session and inferential accuracy, $t(650) = 4.24$, $p < .001$ (see Figure 8B). This indicated that the strength of structural representations was greatest for participants who did not achieve high levels of inference performance (although note that the distance effect was still significant for the majority performance scores).

3. Relatedly, the analysis pointing to neural representation of task structure (as depicted in Figure 7B&C) seems to rely on the existence of a linear relationship between transitive distance and pattern similarity. Conceptually, this makes perfect sense. But, a glance at the data depicted in Figure 7B&C suggests that this linear effect may be driven by a significantly higher similarity for delta 1 pairs relative to the other pairs with no appreciable difference between the delta 2-4 pairs. This coupled with the fact that the strongest effects here come from the recent condition suggest that rather than task structure, participants may instead be only representing the directly experienced premise pairs as more similar with no discernible structure among the other pairs. Is there evidence that these linear relationships truly represent that task structure consistent with the authors claims?

We thank the reviewer for raising this point as we had not previously considered this issue. However, it is not accurate that the linear relationships plotted in Figure 7 are exclusively driven by Δ1 pairs. We now report a set of control analyses that evidence this (see page 15-16):

To validate the distance effects reported above, we ran a series of follow-up tests to examine whether they could be attributed to higher levels of BOLD similarity exclusively for stimuli that were shown within the same premise pair (i.e., driven by Δ1 pairs alone). First, we contrasted similarity estimates between different levels of the distance factor in a random effects analysis. Consistent with our a priori predictions, this invariably revealed significant differences (at uncorrected thresholds) between levels of the distance predictor that did not include Δ1 pairs. We also re-ran each LMM, but excluded similarity estimates between Δ1 pairs. With the exception of the main effect of distance in the hippocampus, all left lateralised effects reported above survived our correction for multiple comparisons, $t(382) > 2.92$.

4. Although I appreciate leveraging MLP simulations to provide computational predictions of the two training schedules, the motivation for this analysis is sparse. Why were MLPs used here, is there prior work that establishes this sort of model as particularly well-suited for predicting human-like transitive inference? Some of this is motivation/framing is provided in the Results section; but I think the authors' approach would be much clearer if the MLP analysis was strongly motivated in the introduction.

As explained above, we have removed the MLP analyses from the main text.

5. I appreciate the inclusion of the descriptive AND and OR models. It would be helpful, however, if connections could be drawn between the descriptive models and the MLP mechanisms. For example, are connection weights between the hidden and output layers in the MLP reflecting OR-type relationships? If possible, connecting these different analyses would strengthen the overall approach.

As we hope is now clear, the MLP analyses were not intended to inform the AND / OR models – they simply provide a motivation for expecting that learning might be better under progressive rather than interleaved training schedules. Since we removed the MLP simulations we do not attempt to connect them with the AND / OR models in this revision.

6. The authors' commitment to open science practices and a priori hypotheses is commendable. However, I wonder if their a priori choice of patterns based on anatomical and/or functional connectivity derived ROIs is limiting the type of neural evidence they can find. For example, the superior and inferior mPFC ROIs from the Schaefer et al. atlas divides this region into two units that don't really correspond mPFC clusters/blobs identified in other studies of structure task representations. Also, in almost all of these ROIs (except for ERC) I wouldn't expect that patterns from the entire extent of the ROI would hold relevant information for the task. A searchlight analysis that considered more localized patterns within the ROIs may be more sensitive to the type of behaviourally related task structure the authors are interested in. I understand that this goes off script and is a clear "exploratory" analysis, but it may provide further insight into the neural mechanisms underlying the clear advantage for progressive training.

We thank the reviewer for raising this issue and agree that the work will benefit from a supplementary analysis based on finer subdivisions of the MPFC. However, because all our effects derive from a set of computationally expensive mixed-effects models, it would not be feasible to run them in a searchlight analysis. Instead, we have reproduced all the imaging analyses on MPFC ROIs derived from a finer whole brain parcellation of the cortex – the Schaefer et al 17-network, 400-region parcellation. The results of these analyses are included in a new supplementary text (S3 Text; see below excerpt from page 27). In short, this supplementary analysis replicated each MPFC effect reported in the main text and identified some additional effects consistent with our a priori predictions.

> *Notably, the MPFC ROIs that we selected for a priori analyses were relatively large compared to the hippocampal and entorhinal ROIs. As such, we provide supplementary analyses of the MPFC based on a finer, 400 cluster, parcellation of the Schaefer et al networks (see S3 Text).*

7. I can infer some of the history in this work from the included response to prior reviewers. And, it is clear that updated analyses may have changed some of the initial findings. But, in its current form, I found that the ROI selection was poorly motivated and the omission of some of these ROIs from the results was confusing. For example, what happened with ERC? I realize that the supplement includes more details about the ROIs and the main text is reserved for the central significant results. But a more thorough description and/or figures that include the results from all ROIs would be help clarify the findings.

We appreciate this point and so now provide further justification of our a priori ROIs (extract from page 27 copied below). Additionally, we have updated all the results tables on the OSF to clarify the findings in each ROI and have included more direct links to this material within the main text.

> *Numerous studies have implicated the hippocampus, entorhinal cortex, and medial prefrontal cortices in memory generalisations [4,7,19,10–17]. As such, our a priori ROIs comprised 8 binary masks that covered all of area in native space (separately in each hemisphere).*

Minor comments

8. Overall, I ran into many typos or missing words. In one place, Figure 9 was referred to but I think should be Figure 8. Apologies that I did not note all of these here, but a careful read and edit of the manuscript is needed.

We thank the reviewer for noting these errors and hope that we have amended each of them.

9. Typo: Second paragraph of Results: "They were not indented...", should be "intended".

Thank you.

10. The first subheading of the Results section doesn't quite reflect the following text as both progressive and interleaved training results are presented. Perhaps the intention here was to refer to the better performance from progressive schedules; if so, the subtitle should be edited to reflect this.

We thank the reviewer for this suggested and have changed this sub-heading to "Interleaved and progressive training in multilayer perceptrons" (now appearing in the supplement).

11. It is not clear what the data points represent in each figure. For example, in Figure 5, each connected pair of data points is presumably relating to a single subject. Figure 4C and D must be representing trial-by-trial RTs. This should be explained in the figure legend.

Thank you for this suggestion; we have now clarified what the meaning of individual datapoints in each figure legend.

12. A complementary paper to the Zhou et al work [43] is by Heffernan et al. 2021 who demonstrated that progressive schedules can be more advantageous in human and model for learning category exceptions. Could be worth including in the current discussion.

We have now included a reference to Heffernan et al in the introduction. However, we have avoided discussing this research in context of other studies on generalisation. The reason for this is that Heffernan et al examined schedule-based facilitation effects related to the acquisition of the very expiation that was being trained. This contrasts with the 'out-of-distribution' generalisation in a transitive inference paradigm. Indeed, a slower learning rate for exceptions can be considered to be better generalisation of the previously acquired rules.

13. I typically consider supplements an open range where authors can include whatever they want. But I was completely befuddled by the inclusion of the successor representation description in Supplemental Figure 4. It is perhaps an interesting point that SRs can represent transitive inference, but this seems like a departure from the current study.

On reflection, we agree with the Reviewer and have removed Supplemental Figure 4.
