# Supplementary material for: Hippocampal and medial prefrontal cortices encode structural task representations following progressive and interleaved training schedules
Source: PLoS Comput Biol. 2022 Oct 17;18(10):e1010566. doi: 10.1371/journal.pcbi.1010566 (PMC9612823; doi:10.1371/journal.pcbi.1010566)
Supplement: Attachment — Submitted filename: Berens-Bird_2022_ResponseToReviewers_R05.pdf [file pcbi.1010566.s010.pdf]

We thank the reviewers who, at all stages, have provided extremely constructive feedback on this manuscript which has substantially improved its quality. In this final round of reviews, we have addressed a couple of minor points raised by reviewer 1. Below we have copied each of the reviewer comments along with are our responses (in bold), and quoted sections of manuscript (indented).

Reviewer 1

The authors have made extensive revisions that appropriately address my previous concerns. I find the new version to be much more streamlined and easier to follow. I appreciate the authors' thoughtful and thorough responses to the previous reviews.

I have a couple of minor remaining points of clarification that the authors may want to address.

1. Pg. 3, bottom: I still think the description of the effects of transitive distance on accuracy could be improved. I appreciate the authors' point that there can be a positive effect in the progressive/remote condition despite being near ceiling for overall accuracy. Is there not also a difference in the effects among interleaved learners, with a stronger effect of transitive distance in the recent condition compared to the remote condition? It may be helpful here to state the effects of transitive distance in each condition separately, or to say more directly that the effect was only significant (rather than "most strongly correlated") in the progressive-remote condition ("t-values < 2.00" suggests this but this is imprecise and easy to overlook, especially when the figure seems to suggest a difference between the interleaved conditions). I press this point because there are differences in the corresponding effects in the subsequent fMRI results, so it seems important to establish what the overall behavioral effects are in each condition.

We agree that this section can be further clarified and so have updated our description of the results in question (see extract from pages 7 and 8 copied below). Specifically, we have now made clear that the effect of transitive distance on performance in the interleaved-recent, interleaved-remote, and progressive-recent conditions are statistically similar. Furthermore, we have adopted the reviewer's recommendation to list the strength of all these effects distinctly.

> *Additionally, there was a significant 3-way interaction between training method, session and transitive distance, t(804) = 3.18, p = .002. This suggested that transitive distance was most predictive of performance in the progressive-remote condition, t(804) = 3.07, p = .002, relative to all other conditions which yielded similar distance effects (t-values = 1.99, 1.08, & 1.55, for the interleaved-recent, interleaved-remote & progressive-recent conditions respectively, see Figure 2B). However, this interaction does not reflect larger performance increases in the progressive-remote condition as the estimated probability of a correct response was uniformly close to ceiling (only increasing from .921 to .996). Instead, it mainly reflects performance becoming more consistently accurate (i.e., lower variances in the binomial probability estimate) with larger distances.*

2. Pg. 22: In the AND/OR model descriptions, what is R_i? Is it supposed to be A_i, or are these different?

We thank the reviewer for raising this question as it highlights a typographical error. In the previous submission, the term $\boldsymbol{R_i}$ was supposed to read $\boldsymbol{A_i}$. This has now been corrected.

Reviewer 2

The revised manuscript and responses from Behrens and Bird offers a comprehensive answer to my initial concerns. Indeed, the authors argument detailing the extent of their effects clarified my original (misguided) concern. Moreover, the control analyses investigating the linear relationship between transitive distance and pattern similarity and the additional look at a more fine-grained parcellation of mPFC provide compelling findings in support of the central claims. Finally, the edits to the revised manuscript (e.g., excluding the MLP analysis and SR simulations) provide a more concise presentation. I appreciate the authors' consideration of the reviewer comments and their detailed reply and control analyses. Overall, this work offers a valuable contribution to the field and I look forward to seeing it published.

We thank the reviewer for extremely helpful and positive reviews.
